# A Prior Usutu Virus Infection Can Protect Geese from Severe West Nile Disease

**DOI:** 10.3390/pathogens12070959

**Published:** 2023-07-20

**Authors:** Hannah Reemtsma, Cora M. Holicki, Christine Fast, Felicitas Bergmann, Martin H. Groschup, Ute Ziegler

**Affiliations:** Friedrich-Loeffler-Institute, Federal Research Institute for Animal Health, Institute of Novel and Emerging Infectious Diseases, 17493 Greifswald-Insel Riems, Germany; hannah.reemtsma@fli.de (H.R.); cora.holicki@fli.de (C.M.H.); christine.fast@fli.de (C.F.); felicitas.bergmann@fli.de (F.B.); martin.groschup@fli.de (M.H.G.)

**Keywords:** flaviviruses, geese, experimental infection, co-protection, Usutu virus, West Nile virus, Germany

## Abstract

Usutu virus (USUV) and West Nile virus (WNV) are closely related pathogens circulating between mosquitoes and birds, but also infecting mammals as dead-end hosts. Both viruses share the same susceptible hosts, vectors, and even distribution areas in Central Europe. The aim of the study was, therefore, to understand their amplification potential and interference upon a successive infection. Two-week old geese were initially infected with an USUV isolate from Germany and with a German WNV isolate17 days later. The geese were susceptible to the USUV and the WNV infections, as evidenced by specific flavivirus antibodies in all of the birds. Furthermore, in half of the USUV-inoculated geese, USUV genomes were detected in the blood and swab samples 2–4 days post-infection. Additionally, most of the examined organs contained USUV genomes and showed signs of encephalitis and ganglioneuritis. Interestingly, upon a sequential infection with WNV, the genome copy numbers in all of the examined samples were significantly lower and less frequent than after a WNV mono-infection. Similarly, the histopathological lesions were less severe. Therefore, it can be concluded that a previous USUV infection can protect birds from clinical disease in a subsequent WNV infection.

## 1. Introduction

The West Nile virus (WNV) and Usutu virus (USUV) are zoonotic pathogens originating from Africa. Both viruses belong to the family *Flaviviridae* and, more specifically, to the Japanese encephalitis virus serocomplex [1]. A serocomplex is defined by the ability of polyclonal post-immune sera against one virus to neutralize other viruses of the same serocomplex [2]. WNV and USUV circulate in a very similar enzootic cycle between susceptible birds as amplifying hosts and mosquitoes, especially *Culex* spp., as vectors [3,4].

USUV was retrospectively detected for the first time outside of Africa in Italy in 1996 [5]. After its first appearance in Austria (2001) [6] and Germany (2010) [7], the virus caused extensive blackbird mortalities [8,9]. In Germany, the first isolate was assigned to the lineage Europe 3 and was restricted to regions north of the Upper Rhine valley and neighboring regions southwest of Germany until 2014 [10]. Thereafter, new introductions of USUV into Germany occurred, followed by a continuous spread [11,12]. Since 2018, USUV has been present throughout the entire country and is divided into five different lineages, namely, USUV Europe 2, 3, and 5, as well as Africa 2 and 3 [13,14]. Taken together, thousands of blackbirds, many captive and wild owls, and other birds have perished in Germany due to USUV infections since 2011 [15]. In humans, there were 110 documented cases of USUV infections in Europe prior to 2022, including 30 with neurological disorders [16]. Therefore, the zoonotic relevance of USUV must not be ignored.

WNV possesses greater zoonotic relevance than USUV, and was detected in Germany in dead birds for the first time in 2018 [17]. In the following years, the WNV lineage 2 strain established itself in eastern Germany and was also found in *Culex pipiens* mosquitoes [18] as potential vectors. Furthermore, WNV has caused the deaths of susceptible birds as well as clinically apparent humans and horses in eastern Germany [19,20,21,22].

Currently, both flaviviruses circulate in overlapping regions in Europe [23,24,25], including Germany [20], as they are transmitted by the same *Culex* genus. However, according to seroprevalence studies in Europe, it is believed that more humans, birds, and mosquitoes are infected with USUV than officially confirmed [26,27,28,29], as USUV does not cause clinical signs as often as WNV.

In recent years, several human co-infections (positive for WNV and USUV by RT-PCRs) have been reported in Austria [29], one in Germany [21], as well as several sequential infections in humans from Italy [30]. Furthermore, in Germany, co-infections have also been described in six birds collected in 2018 and 2019 [31]. The co-occurrence of West Nile virus and Usutu virus genomes in the birds was confirmed by RT-qPCR and genome sequencing [31]. Moreover, recent monitoring studies [20,31,32] of live birds in Germany have revealed very high antibody titers in the virus neutralization tests (VNT) against both viruses. These high antibody titers are most likely not only due to a cross-reaction between the two viruses in the VNT, but possibly also due to sequential or simultaneous infections with both viruses in the same bird [31,32].

Geese are highly susceptible to WNV lineage 1 strains (from Israel 1998 [33] and New York 1999 [34]), which results in high viremia and mortality rates. German domestic geese were also susceptible to WNV lineage 1 from Italy 2009 [35] and WNV lineage 2 from Germany 2018, with detectable viremia levels [36]. Contrarily, according to one study in geese, USUV (Vienna-2001 blackbird, lineage Europe 1) can replicate, but does not result in significant viremia or viral shedding, nor does it cause clinical signs or death in geese [37]. However, the experimental infection of the domestic geese resulted in the seroconversion of the birds 14 days post-infection (dpi) (not studied earlier), with serum titers of hemagglutination inhibition tests from 1:20 to 1:80 [38].

As is well known for mice, a prior infection with a less pathogenic flavivirus (e.g., Zika virus [39] or USUV [40,41,42]) can protect the mice from a lethal WNV infection. In these studies, most of the mice pre-infected with USUV or Zika virus possessed antibodies against the administered flavivirus within 15 days, and these levels rose after the sequential WNV infection. In addition, the pre-infected mice produced detectable WNV specific antibodies very soon (4–5 days) after the WNV infection. However, neither the pre-infection with Zika virus nor that with USUV protected the mice from WNV infection, even though they were protected from severe clinical disease and death [40,41,42]. In magpies, there was a protective effect against a subsequent WNV infection even though neither the USUV genome nor USUV antibodies were found to be present upon WNV infection [43]. To date, no further known studies have focused on USUV and WNV co-infections in amplifying hosts. Therefore, the objective here was to test a possible protective effect of USUV on a subsequent WNV infection in geese.

## 2. Materials and Methods

### 2.1. Geese

For this animal experiment, one day-old geese from the same commercial poultry farm as in the previous mono-infection trial, in Lower Saxony, Germany, were obtained [36]. Throughout the whole experiment, the geese were provided with water and food ad libitum, as well as water basins and hay cobs for enrichment.

### 2.2. Viruses

For the USUV infection, an isolate from a blackbird (*Turdus merula*) from Baden-Wuerttemberg, Germany (2011; GenBank accession no.: HE599647) was used [9]. The virus belonged to the lineage Europe 3 and was titrated on Vero 76 cells (Collection of Cell Lines in Veterinary Medicine, Friedrich-Loeffler-Institut, Greifswald-Insel Riems, Germany), then calculated to have a titer of 10^8.13^ tissue culture infective dose 50 (TCID_50_) per mL [44]. For the WNV infection, the same virus stock as that in the previous mono-infection (WNV lineage 2, GenBank accession no.: MH924836) was used [17,36]. Before the infection, both viruses were diluted in 500 µL minimum essential medium (MEM) to a concentration of 10^6^ TCID_50_.

### 2.3. Procedure of the Animal Trial

**Figure 1:**
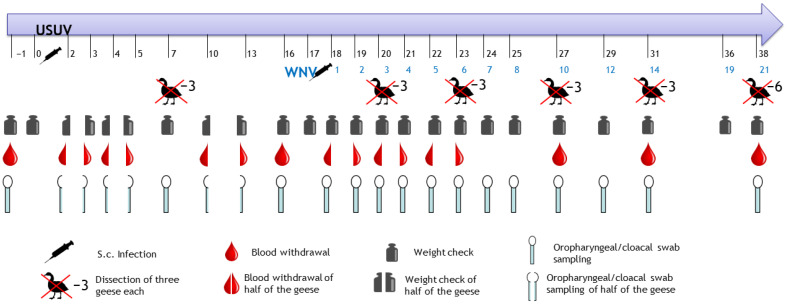
Sampling procedure of the animal trial, s.c.: subcutaneous.

Twenty-one two-week-old domestic geese were infected with 250 µL of virus dilution in each knee fold (Table 1). Subsequently, they were monitored daily by a veterinarian according to a specifically tailored score sheet and additionally checked by video surveillance in the afternoon (Tables S1 and S2) [36]. Three geese were not infected and were kept as environmental controls in a separate cage without any contact with the other groups. Three USUV-infected geese were necropsied (7 dpi USUV), and three geese stayed USUV mono-infected until the end of the experiment (37 dpi USUV). Seventeen days after the USUV infection, the remaining 15 USUV-infected geese were subsequently infected with WNV lineage 2. Table 1 summarizes the allocation of the animal numbers to the experimental procedure and termination dates. Euthanasia of the geese was performed via isoflurane inhalation, followed by exsanguination from the carotid arteries, according to the schedule or upon reaching the humane endpoint (Table 1, Figure 1, Table S1).

### 2.4. Sampling Procedure

Before the infection, all geese were weighed and sampled to ensure that neither flavivirus antigens nor antibodies were present. The sampling included oropharyngeal and cloacal swabs, as well as blood collection from Vena metatarsalis plantaris superficialis. From 2 to 5 dpi USUV, half of the geese were weighed and sampled alternately every second day (Figure 1). At seven dpi with USUV, all of the USUV-infected geese were weighed, and swab samples were taken. At 10 and 13 dpi with USUV, half of the USUV-infected geese were alternatingly weighed and sampled. At sixteen dpi with USUV, all of the USUV-infected geese were weighed and sampled.

After the additional WNV infection (17 dpi with USUV), the sampling scheme was performed identically to that in the WNV mono-infection trial [36]. The three remaining USUV mono-infected geese and controls were weighed and sampled once a week until the termination of the experiment. The processing and RNA extraction of the samples (blood cruors, swab supernatants, and organs) were performed as in the WNV mono-infection trial [36].

### 2.5. Reverse Transcription Quantitative Real-Time Polymerase Chain Reaction (RT-qPCR)

All RNA extracts were examined with both the specific WNV RT-qPCR [45] and the specific USUV RT-qPCR [7]. For the quantification of viral RNA copies in each sample, a calibration curve of synthetic WNV and USUV RNA was run in parallel using 6-fold serial dilutions [45,46]. Additionally, the native virus was diluted, extracted, and used to estimate the TCID_50_/mL by means of an absolute standard curve.

### 2.6. Virus Titration

Samples determined to be positive via RT-qPCR were additionally analyzed by virus titration with an endpoint dilution assay on Vero B4 cells for the WNV-positive samples, and on Vero 76 cells for the USUV-positive samples, respectively. After seven days of incubation, the cells were formalin-fixated and stained with crystal violet, and the virus titers were calculated using the Spearman–Kaerber method [44].

### 2.7. Serology

After heat inactivation (30 min at 56 °C), all of the serum samples were analyzed using virus neutralization tests (VNT) against both viruses [47]: again, for WNV, Vero B4 cells were used, and for USUV, Vero 76 cells. The neutralizing antibody titer (ND_50_) was determined as the reciprocal of the serum dilution that inhibited more than 50% of the cytopathic effects, and was calculated according to the Behrens–Kerber method [48]. Additionally, all plasma samples were examined in the competitive IgG enzyme-linked immunosorbent assay (competition ELISA) (IDvet, Grabels, France) according to the manufacturer’s instructions.

### 2.8. Histopathology and Immunohistochemistry

At necropsy, the tissues (brain, heart, lung, liver, spleen, and small and large intestines, caecal tonsil, kidney, pectoral muscle, and plexus brachialis) were fixated in 4% neutral-buffered formalin for at least 2 weeks prior to dehydration and embedding in paraffin. Sections (3 µm) were prepared and mounted on Superfrost plus slides (Menzel, Darmstadt, Germany). All of the tissues were stained with hematoxylin and eosin.

Additionally, the tissue samples were examined by immunohistochemistry for USUV and WNV using two in-house polyclonal antibodies. Birds routinely submitted to the national reference laboratory and diagnosed with WNV or USUV served as positive controls. Pretreatment included rehydration, inhibition of endogenous peroxidase with 3% hydrogen peroxide (Merck, Darmstadt, Germany) in methanol for 30 min, and a serum block with goat serum alone directly before incubation with the antibody. The in-house OM8 antibody, in a dilution of 1:1700, was utilized for the specific detection of WNV antigens in Proteinase K (Roche, Mannheim, Germany) pretreated (4 µg/mL for 15 min at 37 °C) tissue sections. For the specific detection of USUV antigens, the in-house U433 antibody with a dilution of 1:4000 after a citrate (pH 6) treatment (for 20 min in the microwave) was used. Both antibodies were applied for 2 h at room temperature. The negative control sections were incubated for the same amount of time with goat serum alone. For visualization, the EnVision reagent (Dako Diagnostics, Hamburg, Germany) and diaminobenzidine tetrahydrochloride, counterstained with Mayer’s hematoxylin, were used.

### 2.9. Statistics

The statistical analyses were performed using R (v4.2.2, x64)/R studio (Version 2022.12.0) [49,50]. To compare the WNV viral loads and the WNV antibody titers after the USUV infection to those of the WNV mono-infection [36], a Wilcoxon rank sum test (Mann-Whitney-U-Test) was performed. A *p* value of <0.05 indicated a statistically significant difference. Furthermore, the risk ratio was calculated using the “cohort.count” method to determine at which risk the USUV and subsequently WNV infected geese did not develop WNV antigen or antibodies in comparison to the WNV mono-infected geese. Therefore, all PCR results with copy numbers above zero were considered positive, and those below zero were considered negative. Accordingly, all antibody titers ND_50_ < 1/10 were considered to be negative, and ND_50_ ≥ 1/10 were considered positive.

### 2.10. Ethical Approval

The animal experiment was conducted under biosafety level 3 regulations and, with regard to animal welfare, following national and European legislation—in particular, directive 2010/63/EU. The experiment was approved by the State Office of Agriculture, Food Safety, and Fishery of the federal state of Mecklenburg–Western Pomerania, Germany (LALLF reference number: 7221.3-1-031/20).

## 3. Results

### 3.1. Weight Gain and Clinical Signs

**Figure 2:**
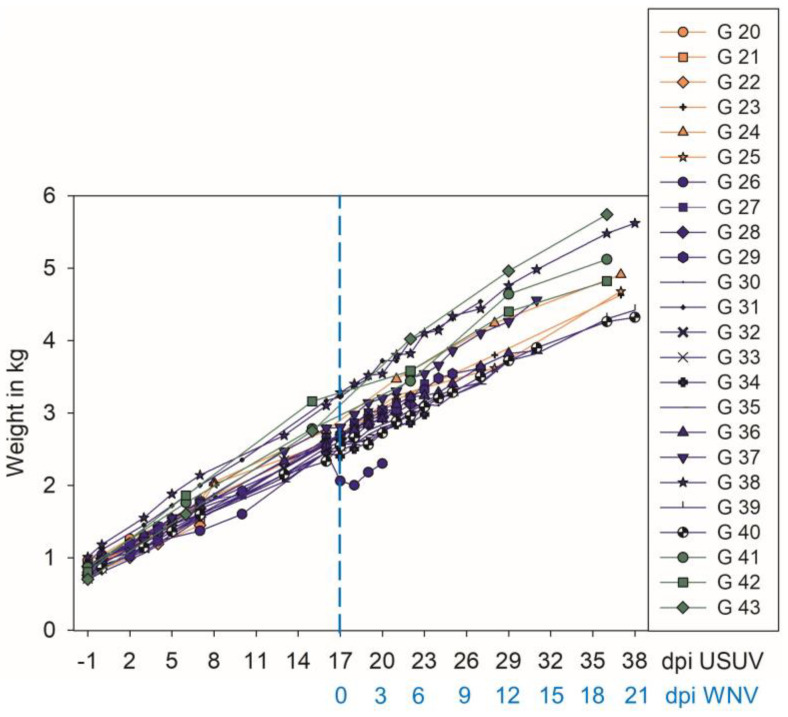
Weight gain of all geese included in the experiment: orange—USUV mono-infected; blue—USUV- and subsequently (17 dpi USUV, dashed blue line) WNV-infected; and green—non-infected controls.

Throughout the 39-day experiment, the animals were monitored daily, as well as weighed and sampled regularly. All of the geese continuously gained weight, except for one goose (G) (number 26) from 16 to 18 dpi USUV (Figure 2). From 7 dpi USUV onwards, this animal showed an orthopedic problem (outward torsion of the leg in the hip joint and walking partially on the tibiotarsal joint) (Table S2). Therefore, it also laid down more frequently. This problem was not apparent before the start of the experiment. Furthermore, another goose showed increased vocalization with no other apparent abnormality (G 25, USUV mono-infected, 18 dpi USUV–28 dpi USUV).

### 3.2. USUV Virological Results

**Figure 3:**
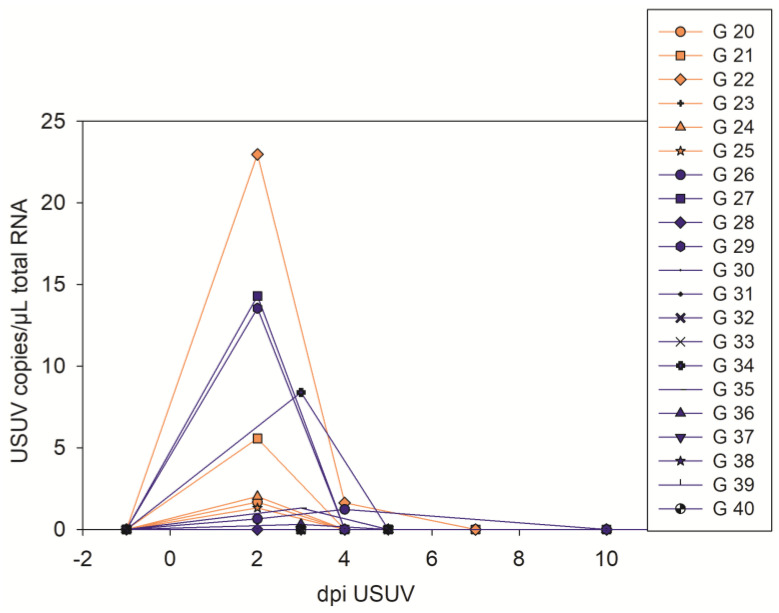
USUV RT-qPCR results of blood cruors of all USUV-infected geese (the orange individuals were USUV mono-infected throughout the experiment, and the blue ones were later additionally infected with WNV).

On day 2 post-USUV infection (day 1: no sampling) most (8/10) of the examined geese were positive in the USUV RT-qPCR of the blood cruors (Figure 3, Table S3). Of these eight geese, in six, replicable virus was also found in their serum (Table S4). At the following sampling points, USUV RNA was only detectable in individual geese, and only at low levels (3 dpi 3/10, 4 dpi 2/11, 10 dpi 1/8).

**Figure 4:**
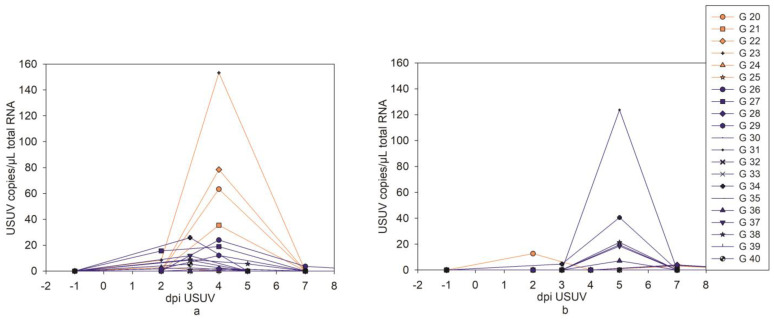
USUV RT-qPCR results of oropharyngeal (**a**) and cloacal (**b**) swab samples of all USUV-infected geese (the orange individuals were USUV mono-infected throughout the experiment, and the blue ones were later additionally infected with WNV).

The majority of the oropharyngeal swabs (Figure 4a, Table S5) were positive in the USUV RT-qPCRs conducted 3 (6/10) and 4 (8/11) dpi USUV. Before and after 3 or 4 dpi, only a few oropharyngeal swabs were positive according to the RT-qPCRs. The largest quantity of positive cloacal swabs in RT-qPCR (Figure 4b, Table S5) was found only 5 dpi USUV (6/10). In addition, isolated cloacal swabs were RT-qPCR-positive from day 2 to 7.

**Figure 5:**
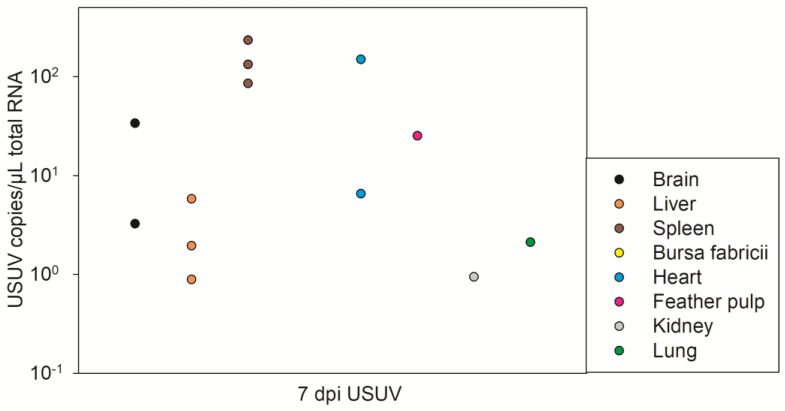
RT-qPCR results of USUV-infected organs of 3 geese at 7 dpi USUV. Negative organs are not shown.

All of the examined organs at 7 dpi USUV were USUV RT-qPCR-positive in at least one of the three individuals, except for the bursa fabricii (Figure 5, Table S6). The highest USUV RNA amounts were measured in all three tested spleens, followed by two hearts, two brains, one feather pulp, and all three livers. The amount of replicable virus in the virus titration was not sufficient to determine a virus titer (Table S7). At 37 dpi USUV, no USUV RNA was detected in the organs of the USUV-infected geese (Table S6).

### 3.3. USUV Pathological Results

**Figure 6:**
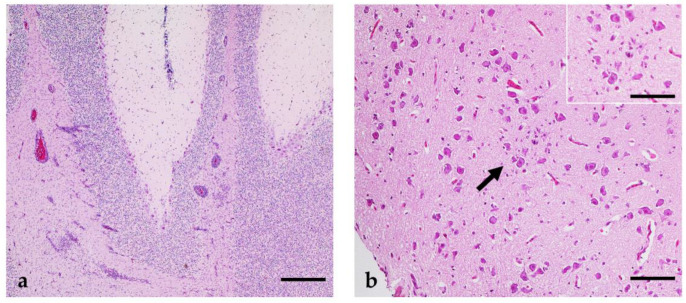
Histopathological lesions seen in geese infected with USUV. (**a**) Cerebellum of G 22 (7 dpi, USUV) with moderate perivascular lymphohistiocytic infiltration, gliosis, and glial nodules; (**b**) cerebrum of G 24 (37 dpi, USUV) with focal mild glial nodule with central single neuronal necrosis. Bars (**a**,**b**): 50 µm (B Inset); 20 µm.

Acute lymphohistiocytic encephalitis (Figure 6a,b, Figure S1) was detectable in four USUV mono-infected geese (4/6). In the geese euthanized at 7 dpi, mild (2/3) to moderate (1/3) lesions were revealed, whereas only one bird (1/3) of the 37 dpi group showed slight signs of a neuroinvasive disease. The cerebrum was the most affected site in all animals, with additional involvement of the brainstem in two geese (2/4) and the mesencephalon in one goose (1/4). Of interest was G 22, which showed marked alterations in the cerebellum (Figure 6a), particularly in the white matter and extending into the granular layer. The histopathological characteristics were the same in all birds and included perivascular lymphohistiocytic cuffing with lymphocytolysis, scattered glia nodules (2/4, Figure 6b), and endothelial hypertrophy (2/4). In addition, a multifocal widespread acute lymphohistiocytic ganglioneuritis of the enteric nervous system (ENS) was diagnosed in all geese at 7 dpi (3/3), and in two of these animals, this was associated with signs of neuronal degeneration of single cells. Moreover, two geese also showed corresponding lesions in a vegetative ganglion attached to the loose connective tissue of the gut. The gut walls (*Tunica muscularis*, *subserosa*) of these animals (3/3) also showed corresponding multifocal perivascular cuffing, which is only rarely seen. In the 37 dpi group, these alterations were seen less frequently, with mild inflammatory cell infiltrations in the *Plexus myentericus* (1/3) and *Tunica muscularis* (2/3).

The spleens of almost all birds (3/3 at 7 dpi and 2/3 at 37 dpi) revealed follicular hyperplasia with central lymphocytolysis and intrafollicular detection of tingible body macrophages.

Additional findings were an oligofocal slight (1/3 at 37 dpi) up to mild (2/3 at 7 dpi) acute lymphohistiocytic myocarditis, in one case with slight focal signs of necrosis (7 dpi). At 7 dpi, one animal (1/6) showed acute lymphohistiocytic myositis (*Musculus* (*M*.) *pectoralis*). A multifocal mild subacute to chronic lymphoplasmacytic pericholangitis was seen in two animals at 7 (1/3) and 37 (1/3) dpi, whereas an additional bird (1/3, 37 dpi) revealed signs of a chronic-active (lymphiohistiocytic and lymphoplasmacytic) pericholangitis. Additionally, one animal (1/6) showed signs of fatty hemorrhagic liver syndrome upon gross examination at 37 dpi.

The most important histomorphological findings are summarized in Tables S8 and S9. No USUV antigens were detectable by immunohistochemistry in the either 7 dpi USUV or 37 dpi USUV organs.

### 3.4. WNV Virological Results

**Figure 7:**
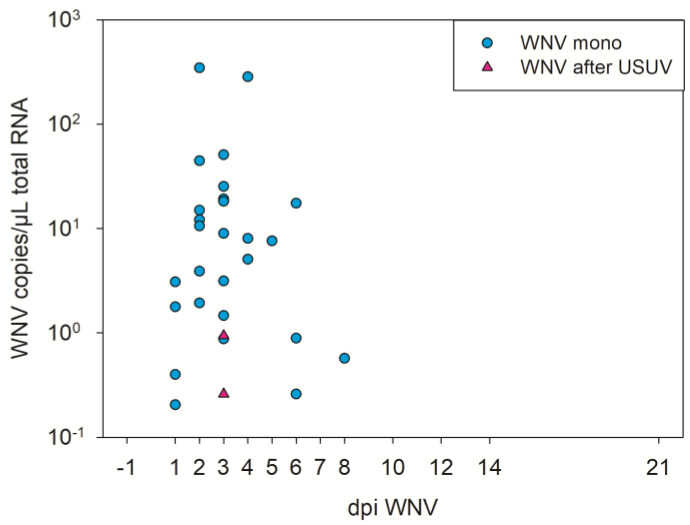
Comparative illustration of RT-qPCR results of the blood cruors from WNV mono-infected geese (from a previous experiment, published in [36]) in blue dots, and from USUV and subsequently WNV-infected geese in pink triangles. Both the total number of geese and the number of geese tested per day were similar in both experiments, and negative results are not depicted.

After the following WNV infection, only very low levels of WNV RNA were detected in the blood cruors of two of the geese (2/9) at 3 dpi WNV (Figure 7, Table S10). This corresponded to a statistically significant difference from the blood cruor genome copies of the WNV mono-infected geese obtained from a previous experiment (1–3 dpi *p* value < 0.05, Table S11) [36]. Furthermore, the risk ratio of the WNV infected geese after USUV infection to not have viral copies in their blood was higher as for the WNV mono-infected geese (1–10 dpi: risk ratio (RR) >1, Table S11).

**Figure 8:**
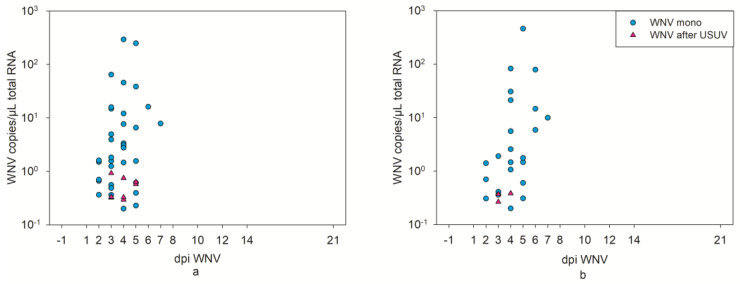
Comparative illustration of WNV RT-qPCR results of the oropharyngeal (**a**) and cloacal (**b**) swab samples from WNV mono-infected geese (from a previous experiment [36]) in blue dots, and from WNV-infected geese after USUV infection in pink triangles. Both the total number of geese and the number of geese tested per day were similar in both experiments, and negative results are not depicted.

WNV RNA was also barely detectable in the swab samples (2–4 WNV-positive oropharyngeal swabs from 3–5 dpi WNV, and 2 and 1 WNV-positive oropharyngeal swabs at 3 and 4 dpi WNV, respectively) (Figure 8, Table S12). Again, this indicates a statistically significant difference in virus genome detection in swabs of the WNV mono-infected geese (2–5 dpi *p* value oropharyngeal < 0.05, 4–6 dpi cloacal < 0.05, Table S11). Also, the ratio of the WNV infected geese without viral copies in their swabs after USUV infection was higher than for the WNV mono-infected geese (2–7 dpi RR oropharyngeal and cloacal > 1, Table S11).

**Figure 9:**
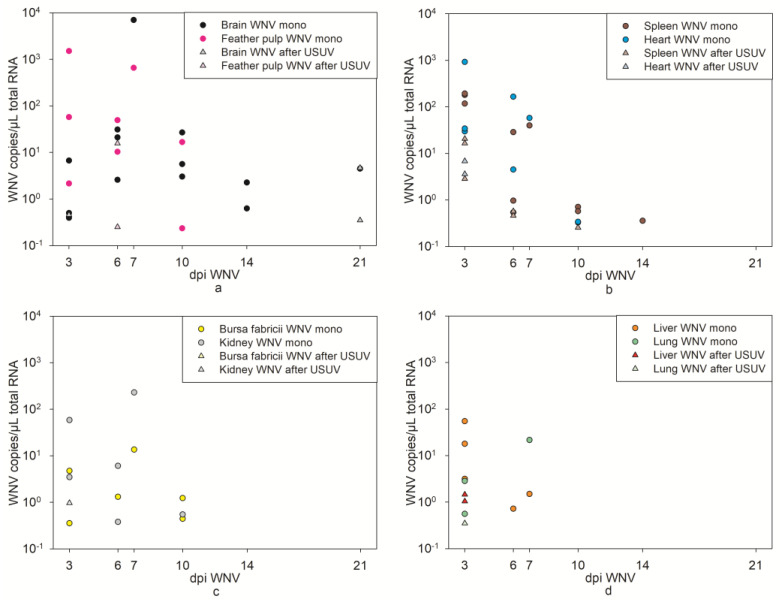
Comparative illustration of WNV RT-qPCR results of organs from WNV mono-infected geese (from a previous experiment [36]) in dots, and from USUV- and subsequently WNV-infected geese in triangles. (**a**) Brain and feather pulp; (**b**) spleen and heart; (**c**) bursa fabricii and kidney; and (**d**) liver and lung samples. In each case, 3 animals were analyzed per time point. Organs without detectable WNV RNA are not depicted. “mono“ refers to the results from the previous WNV mono-infection [36] and “WNV after USUV“ refers to the results of the WNV infection after recent USUV infection.

At 3 dpi, low WNV copy numbers were detected in the majority of the spleens, hearts, and livers. In the brains, lungs, and kidneys, a very low viral copy number was detected in only one goose (1/3) (Figure 9, Table S13). At 6 dpi, very low WNV virus copies were present in only two of the spleens and feather pulps. Interestingly, at 21 dpi, two brains were WNV-positive according to RT-qPCR. When taking all organs together, as well as all organs separately (except the liver and lungs), the copy numbers differed statistically significantly from those of the previous WNV mono-infection (*p* value < 0.05, Table S11). Again, the organs (except for the bursa fabricii) of the WNV-infected geese after USUV infection had a higher risk ratio of not having WNV copies in their organs than the WNV mono-infected geese (RR > 1, Table S11). Furthermore, the quantity of replicable virus in the virus titration was not sufficient to determine a virus titer (Table S7).

All control samples were negative in both of the RT-qPCRs at all of the examined time points. In addition, the samples of the USUV mono-infected geese were negative according to the WNV RT-qPCR (Tables S10, S12 and S13). Correspondingly, after the WNV infection, all samples were negative according to the USUV RT-qPCR (Tables S3, S5, and S6).

### 3.5. WNV Pathological Results

**Figure 10:**
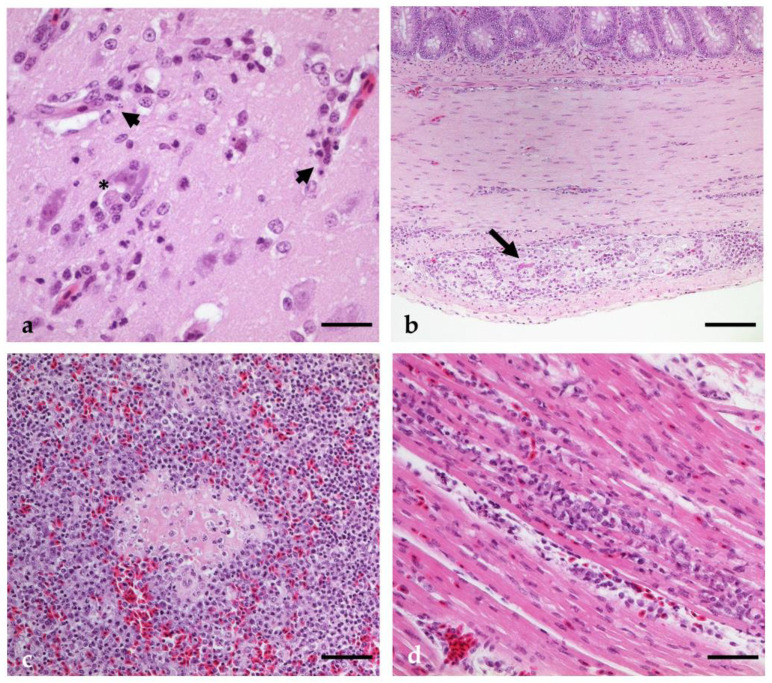
Histopathological lesions seen in geese infected with USUV and subsequently with WNV. (**a**) Cerebrum of G 40 (38 dpi USUV/21 dpi WNV) with mild perivascular lymphohistiocytic infiltration extending into the neighboring neuropil and focal satellitosis (*); note the signs of lymphocytolysis (arrowheads). (**b**) Small intestine of G 27 (23 dpi USUV/6 dpi WNV) with moderate lymphohistiocytic infiltration and signs of neuronal degeneration (arrow). (**c**) Spleen of G 32 (20 dpi USUV/3 dpi WNV) with focal fibrinoid necrosis of the periarteriolar lymphoid sheath. (**d**) Heart of G 33 (20 dpi USUV/3 dpi WNV) with focal mild lymphohistiocytic myocarditis. Bars: (**b**) 50 µm, (**c**,**d**) 20 µm, (**a**) 10 µm.

A multifocal subacute lymphohistiocytic encephalitis (Figure 10a) was seen in almost all geese (12/15), from a slight (3/12) to a mild (5/12) or moderate (4/12) degree, whereas one goose only revealed single scattered glia nodules. It is noteworthy that the three birds (3/4) with the most pronounced lesions were found late in the incubation period (14–21 dpi) (Figure S1). The distribution of lesions in the brain was variable; however, the cerebrum was involved in most cases (10/12), followed by the brain stem (6/12), cerebellum (5/12), and mesencephalon (3/12). A widespread distribution involving more than one brain region was mostly seen in animals with more pronounced alterations (3/4). Furthermore, in two geese (2/12), the lesions were confined to the brain stem. The most prominent histopathological lesions consisted of oligo-/multifocal perivascular lymphohistiocytic perivascular cuffing (12/12), which is frequently associated with signs of lymphocytolysis (7/12). Plasma cells were rarely involved (4/12), and were only found in geese of the 14 and 21 dpi groups (3/4). Glial nodules were frequently observed (10/12), whereas isolated necrosis of single glial and neuronal cells (5/12), satellitosis (4/12), and endothelial hypertrophy (3/12) were rare.

Five geese (5/15) showed multifocal acute slight (2/5), mild (2/5), or moderate (1/5) lymphohistiocytic ganglioneuritis of the ENS with neuronal degeneration of single cells (Figure 10b), accompanied by corresponding inflammatory cell infiltration into the gut wall (*Tunica muscularis*, *subserosa*). Interestingly, all affected geese were found in the first half of the incubation period, at 3 dpi (1/3), 6 dpi (3/3), and 10 dpi (1/3). Inflammatory cell infiltration confined to the gut wall without affecting the ENS was also seen at later time points (4/6); however, in most cases, this was observed only to a minor extent (3/4). One goose had a more pronounced inflammatory reaction, but this goose also had a focal granuloma in the wall of the proventriculus.

The spleens of almost all of the birds (10/15) were characterized by follicular hyperplasia with central lymphocytolysis and intrafollicular detection of tingible body macrophages. A slight increase in histiocytes in the sinuses was seen in seven animals (7/15). Mild oligofocal fibrinoid necroses of the periarteriolar lymphoid sheaths (Figure 10c) were detectable in three geese (3/15).

Oligofocal slight (6/9) to mild (3/9) acute lymphohisticocytic myocarditis (Figure 10d) was frequently observed (9/15); however, only 3 geese from the 3 (1/3) and 6 dpi (2/3) groups revealed slight signs of necrosis of isolated muscle fibers.

Rare findings were oligofocal mild acute lymphohistiocytic myositis (*M. pectoralis*) in four birds (4/15), focal mild acute lymphohistiocytic neuritis (2/15), and oligofocal acute lymphohistiocytic interstitial nephritis (2/15).

The livers displayed variable alterations, including signs indicative of fatty hemorrhagic liver Syndrome (mainly subcapsular hemorrhages associated with acute hepatocellular necrosis, biliary duct proliferation, and fibroangioblastic granulation tissue) in 12 animals (12/15). Several birds were also diagnosed with multifocal subacute mild pericholangitis (3/15) or cholangitis (1/15). Intralobular mild lymphohistiocytic hepatitis was also detectable in a few animals (6/15), and slight signs of hepatocellular necrosis were seen, albeit rarely (2/6).

The most important histomorphological findings are summarized in Table S8 and S9, and are compared to the findings of the previous experiments in Table S1.

Through immunohistochemistry, neither USUV nor WNV antigens were found at any of the examined time points.

### 3.6. Controls’ Pathological Results

None of the control geese showed alterations indicative of a viral infection. However, during gross examination, two animals (2/3) portrayed signs of fatty hemorrhagic liver Syndrome. Moreover, all geese (3/3) revealed multifocal mild subacute to chronic pericholangitis associated with distinct biliary duct proliferation. One animal (1/3) additionally showed focal mild acute lymphohistiocytic interstitial nephritis.

### 3.7. Serology

**Figure 11:**
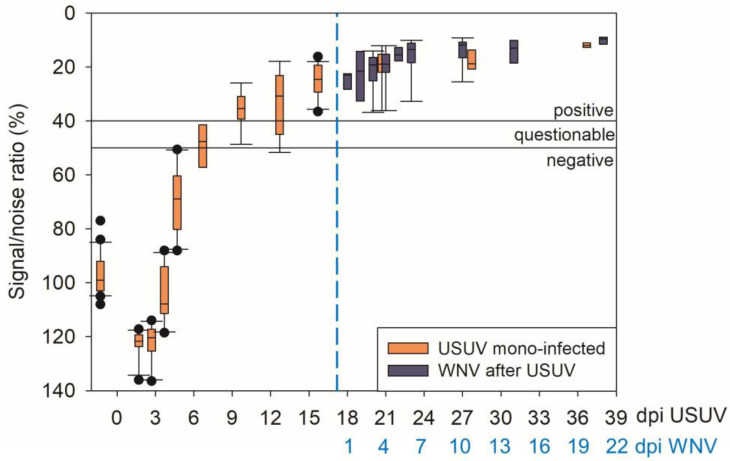
Comparative illustration of flavivirus-specific IgG ELISA results of all infected geese: in orange, USUV mono-infected; in blue, USUV- and subsequently WNV-infected (17 dpi USUV, dashed blue line).

In the IgG ELISA, all but one examined blood plasma sample from the USUV-infected geese at 10 to 19 dpi were flavivirus-positive or questionable (Figure 11, Table S14). From 20 dpi onwards, all plasma samples from both groups (USUV mono-infected and USUV- and subsequently WNV-infected) were flavivirus-positive; therefore, all birds seroconverted.

**Figure 12:**
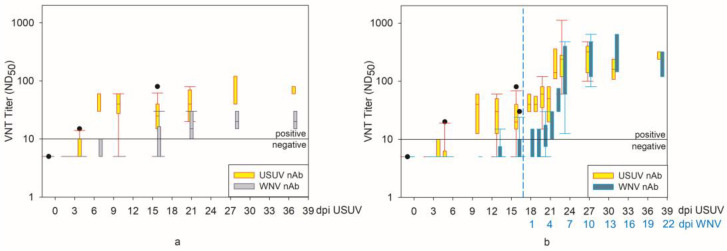
VNT results of (**a**) the USUV mono-infected geese in the USUV VNT in yellow and the WNV VNT (cross-reactive) in gray; (**b**) USUV- and subsequently (17 dpi USUV, dashed blue line) WNV-infected geese in the USUV VNT in yellow and the WNV VNT in cyan.

In the USUV-specific VNT (Figure 12, Table S15), the first USUV-neutralizing antibodies (nAbs) were detectable in the sera of the USUV-infected geese at 4 dpi. At 10, 13, and 16 dpi, USUV nAbs were found in all tested sera with one exception per time point. They reached their maximum values at 28 and 37 dpi USUV (Figure 12a). At 20 dpi USUV, 3 days after the infection of the geese with WNV, the USUV nAb levels were re-boosted in the corresponding geese (Figure 12b). One day later, the WNV serum titers of these geese also steadily began to increase (4 dpi WNV/21 dpi USUV), and remained at a plateau until the end of the experiment.

Interestingly, at 7 dpi with USUV, individual sera from the USUV mono-infected geese were able to neutralize not only USUV, but also WNV, in the WNV VNT (cross-reactive at lower titers; Table S15). This explains why the WNV nAb titers were significantly higher in these geese than in the WNV mono-infected geese of the previous experiment both prior and shortly after the WNV infection (-1–6, except 4, dpi WNV; *p* value < 0.05, RR < 1, Table S11 [36]). Towards the end of the experiment, the WNV and USUV nAb titers approached one another and were surprisingly similar to those of the WNV mono-infected geese of the previous experiment (Figure 12; Tables S11 and S15) (Tables S14 and S15).

## 4. Discussion

The primary aim of this study was to examine the pathogenesis of WNV after a previous USUV infection and to clarify the potential protective effect of successive flavivirus infections. An additional goal of the study was to determine the overall susceptibility of geese to USUV. Thus, samples were taken regularly from the geese not only after the WNV infection, but also previously, during the course of the USUV infection.

The geese not only were susceptible to USUV, as illustrated by seroconversions in all birds, but the majority also had viral copies in their blood and swab samples. This is in contrast to the experiment reported by Chvala et al., which only found USUV RNA in the plasma of one goose (1/11), even though the intramuscular route of inoculation should have facilitated an infection [37]. However, a different virus isolate (Europe 1) with a lower infectious dose (5 × 10^4^ TCID_50_/0.5 mL) was used in this earlier study, which certainly had an impact on the USUV infection efficiency in the geese.

In the study presented herein, the organs of the USUV-infected geese were only examined at 7 and 37 dpi with USUV (3 geese per time point), because the main focus was placed on the subsequent WNV pathogenesis. As expected, the highest viral loads were found at 7 dpi, with the spleen, heart, and brain being the most affected organs. However, whereas in the histopathology, distinct encephalitis was found in all of the birds at this time point, viral loads were confined to two birds, indicating a prolonged inflammatory reaction even after the initial clearance of the virus. This theory is also supported by the results seen at 37 dpi: viral RNA was not detected in brain samples, but one animal still showed slight signs of acute lymphohistiocytic encephalitis. These results demonstrate that USUV successfully infected the geese by causing viremia and spreading to lymphatic organs, such as the spleen. Moreover, the distinct ganglioneuritis shown in the ENS early rather than late in the incubation period clearly indicates a crucial role of the vegetative nervous system in the early pathogenesis of the disease, as was already discussed for WNV [33,36]. Hence, USUV infection must also be added to the list of possible differential diagnoses associated with acute non-suppurative ganglioneuritis of the ENS in birds. The lesions found in the brains of geese were largely consistent with previous studies in various avian species [51,52], but it is noteworthy that the necrotic component was absent not only in the brains, but also in hearts and spleens, of the geese. Both organs were mildly and nonspecifically affected, and viral antigens could not be found. This very mild course of infection (compared to WNV) was also noted by Chvala et al., who demonstrated glial nodules in only 1 out of 11 infected geese [37]. In summary, the viremic load in geese was not sufficient to re-infect American *Culex* spp. with USUV [53]. Nonetheless, geese are suitable sentinel species for the monitoring of flavivirus infections, since they seroconvert quickly (persistently over 37 days), and WNV and USUV antibodies can be distinguished by VNTs.

Seventeen days after the USUV infection, 15 geese were additionally infected with WNV lineage 2 from Germany. The same WNV isolate had already been used for a WNV pathogenesis study (mono-infection) in domestic geese, which was presented previously [36], allowing for a direct comparison of the results. In the previous study, WNV mono-infected geese showed viremia up to 10^5.5^ TCID_50_/mL, viral shedding, and viral loads in the organs up to 10^6.0^ TCID_50_/mL in organ suspension. These high WNV titers, especially in one goose, could have been sufficient to sustain the natural WNV transmission cycle [36,54,55]. Moreover, in the WNV mono-infected geese, WNV antigens were found by immunohistochemistry as well as distinct histopathological lesions indicative of a viral infection [36]. Interestingly, in the study presented herein, only two of the USUV- and subsequently WNV-infected geese had detectable WNV RNA copies in their blood at 3 dpi with WNV, and there were also low levels of WNV RNA copies found in a few of the swab samples. Also, in the organs of the USUV- and subsequently WNV-infected geese, only small numbers of WNV-RNA copies were detectable in a few of the geese, which were mainly confined to the spleens. Therefore, most of the examined organs from the USUV- and subsequently WNV-infected geese differed significantly from those of the WNV mono-infected geese. Exceptions were the lungs and livers, which already contained only a few RNA copies when mono-infected with WNV. The overall milder disease course observed in most of the birds compared to the previously published WNV mono-infection [36] is consistent with human cases, in which WNV infections, secondary to USUV infections, have been shown to be asymptomatic or to exhibit only mild symptoms [30]. This is also corroborated by the results of the histopathological examination in the present study; neither encephalomalacia nor necrotic myocarditis nor lesions of a systemic vasculitis were detectable in the geese. Moreover, while a clear time dependence in the course of the disease was observed after the WNV mono-infection (and the USUV infection presented herein), with a peak between 6 and 14 dpi, this was not observed with the sequential USUV and WNV infections. On the contrary, the observed variability was more indicative of individual differences between the animals, indicating an individually different degree of cross-protection resulting from the previous USUV infection.

It is known from studies on immune-deficient transgenic mice that macrophages [56] and CD8^+^ T cells [57] play an important role in protection against neuroinvasive WNV infections. Therefore, it is remarkable that, despite the high nAb titers and, most likely, also the cross-protective T cells, brain entry was mostly not prevented. Even more interesting, however, is that most of the geese with moderate encephalitis were detected in the second half of the incubation period (14–21 dpi). Hence, the assumed cellular cross-protection may subside as the disease progresses, resulting in more severe brain lesions later on. The extent to which this is a self-perpetuating immunopathological process or a latency of the pathogen that triggers the inflammatory processes with a time delay once the cross-protection wears off cannot be finally resolved. The number of animals investigated herein is too low to allow general conclusions to be drawn. Further studies addressing the still-predominant lymphohistiocytic perivascular infiltration would, therefore, be of interest. It also must be kept in mind that the brain lesions observed at earlier time points after the WNV infection (3–10 dpi) may have been originally induced by the previous USUV infection. This theory is additionally supported by the fact that WNV-RNA and viral antigens were only rarely detected in the brains of these animals. Therefore, it is tempting to speculate that WNV replication in the central nervous system was delayed due to the previous USUV infection. In this regard, it is also of interest that clear signs of ganglioneuritis were also observed in the additionally WNV-infected animals during the first half of the incubation period, as was also seen in the USUV and WNV mono-infected animals [36]. This clearly indicates that, in contrast to the neuroinvasion of the brain, potential early replication of WNV in the ENS appears not to be affected by possible cross-protection and may serve as a latent source of infection.

The protective effect of a prior USUV infection, especially in regard to WNV clinical signs and viral loads (in blood, swabs, and organs), can be attributed to the presence of flavivirus nAbs after 10 dpi with USUV. As WNV and USUV are closely related and share antigenic characteristics with their structural outer proteins, they belong to the same serogroup [58]. The main target of nAbs for most of the members of the flavivirus genus is the E protein [59]. This E protein is involved in the entry of flaviviruses into the host cells [60], and is the main virulence factor of WNV [61] and USUV [41]. As was reviewed by Vaughan et al., antibody-mediated immunity is considered an important factor in the protection against flavivirus (re-)infections [62]. According to the definition of a serogroup, sera are able to neutralize other viruses of the same serogroup after infection with one virus [2]. This was evident in this study, as at 10 dpi USUV, nearly all of the USUV-infected geese had USUV nAbs, which could additionally neutralize WNV at 13 dpi USUV (ND_50_ titers above or equal to 1:10). Furthermore, at 16 dpi USUV, all geese had IgG antibodies, as was detected using the ID Screen^®^ West Nile Competition Multi-species ELISA directed at the pr-envelope (E) antigen.

The “gold standard” for the differentiation of flavivirus antibodies is plaque-reduction neutralization tests or virus neutralization assays in microplates [63]. Even though the same virus isolates were used for the VNTs in this study as well as for the infection of the geese, cross-reactions were observed in the VNTs, especially when analyzing the serum of the USUV mono-infected geese. After 16 dpi USUV, some of the sera were cross-reactive against WNV, with WNV nAbs titers higher than one-quarter of the USUV nAbs titers. However, the ratio of USUV nAbs to cross-protective WNV nAbs of the USUV mono-infected geese was lower than the ratio of WNV nAbs to the cross-protective USUV nAbs of the WNV mono-infected geese [36]. Also, for Eurasian coots (*Fulica atra*), it was suspected that the antibody response to USUV was not as strong and long-lasting as the response to WNV [64]. One possible explanation could be that USUV is less pathogenic for geese, resulting in lower antigen production. Therefore, the antibody response against USUV is lower in the USUV mono-infected geese than the WNV nAb titer in the WNV mono-infected geese [36].

Furthermore, of particular interest is antibody development after the WNV challenge: at 4 dpi with the WNV challenge (21 dpi USUV), the USUV nAbs increased first. Only in the following days (5–6 dpi WNV) did the WNV nAbs then increase. This increase in USUV nAb after WNV infection is due to the so-called antigenic sin. This means that secondary exposure to a divergent, non-identical antigen is not recognized as such by the B cells (or cytotoxic T lymphocytes), and the immune system relies on its "memory" of the original, primary antigen to develop a response to the variant [65]. Thus, USUV nAbs of the USUV mono-infected geese were, in the end, significantly lower than those of USUV- and subsequently WNV-infected geese. Comparable results already exist for mice: after vaccination with WNV-recombinant subviral particles, mice induced low IgG levels, which were cross-reactive with USUV and were boosted after the challenge with USUV [66].

Although protection against a flavivirus infection is thought to rely primarily on the humoral immune response [62], the cellular immune response may also play a beneficial role. Magpies that had been experimentally infected with USUV without developing viremia, detectable viral replication in organs, or eliciting USUV nAbs, for example, were protected from a subsequent and usually fatal WNV infection [43]. Also, in adult mice, neither USUV RNA nor USUV nAbs were detected after an USUV infection [40]. After the subsequent WNV challenge, mice sera neutralized both viruses with higher titers against WNV, and most of the mice survived the WNV challenge [40].

It must be kept in mind that a limitation of this study is that all three infections (WNV-mono-infection, WNV infection after USUV infection, and USUV mono-infection) were performed in geese of different ages. This was due to animal welfare reasons, as the main aim of this study was to demonstrate a co-protection between USUV and WNV. It is known from experimental WNV infections and WNV outbreaks that the age of a bird has an impact on its susceptibility to a flavivirus infection [34,67].

Furthermore, under field conditions, it is not always a given that successive infection takes place about three weeks after the initial infection. Much more often, the interval will be longer. Nevertheless, it can be assumed that even with longer intervals between infections, there is a protective effect of a severe WNV infection in terms of clinical symptoms, histomorphological changes, and viremia.

As birds constitute as amplifying hosts for WNV, this effect could also slow down the WNV transmission cycle as fewer mosquitoes come in contact with infectious birds. Neutralizing antibodies produced following a flavivirus infection are thought to be long-lasting; however, it is unknown how long the cross-protective antibodies (against a heterologous flavivirus) may last [68]. Nevertheless, the pre-existence of USUV in Europe could help to explain why WNV did not spread as explosively in Central Europe as it did in North America, where the newly introduced virus encountered an immunologically naïve bird population. Currently, in Europe, and especially in Germany, annual outbreaks of USUV (since 2011) and WNV (since 2018) occur, the extent of which depends mainly on environmental factors, i.e., warm springs and hot summers. However, to date, WNV outbreaks in Germany have been limited to eastern Germany and have only occurred on a small scale, with cases in birds as well as in horses and humans [20,22]. Further studies are needed to fully understand the interference and immunological aspects of USUV and WNV infections in birds.

## 5. Conclusions

In summary, an infection with a German USUV isolate is milder in geese than an infection with a German WNV isolate, but with comparable viral copy numbers. The WNV infection following the USUV infection showed significantly reduced viremia, viral excretion, and viral loads, as well as decreased histopathological alterations in the organs, compared to the WNV mono-infection. These results indicate a protective effect against severe WNV disease, and can be attributed to cross-reacting WNV nAbs that were already present before the WNV infection.

## Figures and Tables

**Table 1:** Number of geese and infections, as well as necropsy time points.

Goose Number	Virus 1	Virus 2	Date of Euthanasia
G 20–22	USUV	No virus	7 dpi USUV
G 23–25	USUV	No virus	37 dpi USUV
G 26, 32, 33	USUV	WNV	20 dpi USUV and 3 dpi WNV
G 27, 28, 34	USUV	WNV	23 dpi USUV and 6 dpi WNV
G 29–31	USUV	WNV	27 dpi USUV and 10 dpi WNV
G 35–37	USUV	WNV	31 dpi USUV and 14 dpi WNV
G 38–40	USUV	WNV	38 dpi USUV and 21 dpi WNV
G 41–43	No virus/controls	No virus/controls	36 d after start of the experiment

dpi: days post-infection; USUV: Usutu virus; WNV: West Nile virus. Counting of the geese (G) started at G 20 to allow for a comparison with geese (G 1–G 18) from the previous WNV mono-infection [36].

## Data Availability

The data that support the findings of this study are available in the main manuscript and in the Supplementary Materials for this article.

## Supplementary Materials

The following supporting information can be downloaded at: https://www.mdpi.com/article/10.3390/pathogens12070959/s1, Table S1: Score sheet as an assessment criterion for the health status of the animals; Table S2: Clinical scores of geese (G), infected with USUV and/or WNV via subcutaneous injection; Table S3: Results of USUV RT-qPCR of blood cruor of all infected geese, indicated as Ct values and copies/µL total RNA; Table S4: Results of serum titration of RT-qPCR-positive blood cruors in TCID50/mL; Table S5: Results of USUV RT-qPCR of swabs of all infected geese, indicated as Ct values and copies/µL total RNA; Table S6: Results of USUV RT-qPCR of organ homogenates of all infected geese, indicated as Ct values, copies/µL total RNA, and copies/mg original organ; Table S7: Results of titration of RT-qPCR-positive organ homogenates in TCID50/mL; Table S8: Overview of the degree and distribution of the most important histopathologically detectable alterations in the USUV mono-infected geese (G 20–25), the USUV- and subsequently WNV-infected geese (G 26–40), and the controls (G 41–43); Table S9: Overview of the degree and distribution of the most important histopathologically detectable alterations in the central nervous system of the USUV mono-infected geese (G 20–25), the USUV- and subsequently WNV-infected geese (G 26–40), and the controls (G 41–43); Table S10: Results of WNV RT-qPCR of blood cruors of all infected geese, indicated as Ct values and copies/µL total RNA; Table S11: *p*-values of Wilcoxon rank sum test for comparing the WNV viral loads and the WNV antibody titers after previous USUV infection with those after a former WNV mono-infection; Table S12: Results of WNV RT-qPCR of the swab samples of all infected geese, indicated as Ct values and copies/µL total RNA; Table S13: Results of WNV RT-qPCR of organ homogenates of all infected geese, indicated as Ct values, copies/µL total RNA, and copies/mg original organ; Table S14: Results of flavivirus-specific IgG ELISA of all geese; Table S15: Results of WNV and USUV virus neutralization tests from all tested sera; Figure S1: Comparative presentation of the histopathological necroses (grades from 0.0 not shown to a maximum of 3.0) in different tissues. Three geese each from the group of WNV mono-infected animals and WNV-infected animals after a recent USUV infection were examined at 3, 6, 10, 14, and 21 days post-infection (dpi). Additionally, three USUV-mono-infected geese were evaluated at 7 and 37 dpi.
