# Supplementary material for: A Prior Usutu Virus Infection Can Protect Geese from Severe West Nile Disease
Source: Pathogens. 2023 Jul 20;12(7):959. doi: 10.3390/pathogens12070959 (PMC10386565; doi:10.3390/pathogens12070959)
Supplement: Supplementary file 1 [file pathogens-12-00959-s001.zip › Figure S1.pdf]

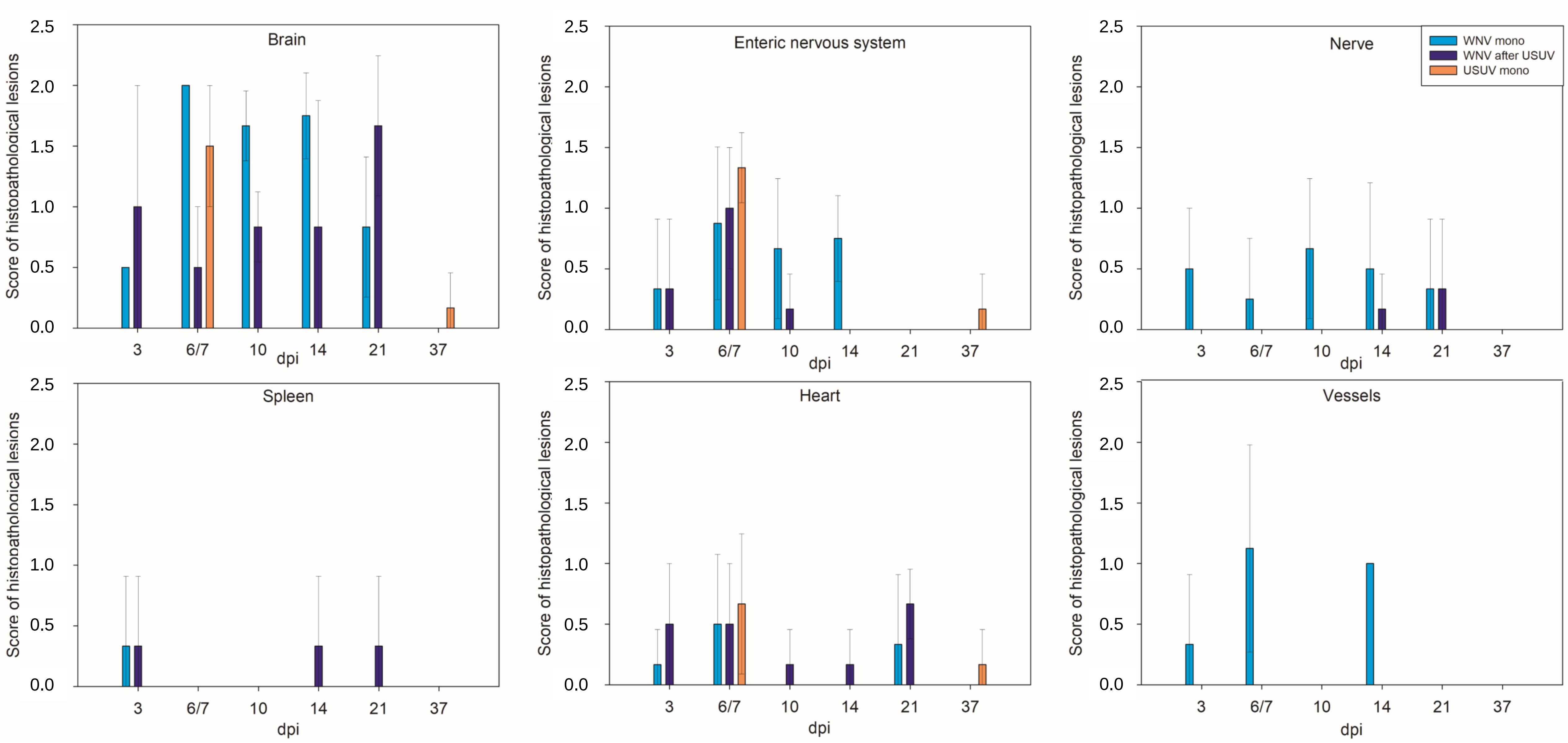

Figure S1. Comparative presentation of the histopathological necroses (graded from 0.0 not shown to a maximum of 3.0) in different tissues. Three geese each from the group of WNV mono-infected animals and WNV infected animals after a recent USUV infection were examined 3, 6, 10, 14, and 21 days post infection (dpi). Additionally, three USUV-mono-infected geese each were evaluated 7 and 37 dpi.
